# Clinical Features of Fatal Pandemic Influenza A/H1N1 Infection Complicated by Invasive Pulmonary Fungal Infection

**DOI:** 10.1007/s11046-019-00421-z

**Published:** 2019-12-27

**Authors:** Zhuxi Yu, Qin Gu, Beiyuan Zhang, Xiancheng Chen, Jian Tang, Yayi Hou, Wenkui Yu

**Affiliations:** Nanjing University Medical School Affiliated Nanjing Drum Tower Hospital, Nanjing, China

**Keywords:** H1N1, Human leukocyte antigen-DR isotype (HLA-DR), Invasive pulmonary fungal infection, Severe pneumonia

## Abstract

**Background:**

Severe pneumonia caused by influenza virus infection can be secondary to invasive pulmonary fungal (IPF) infection.

**Objectives:**

This study aimed to summarize the incidence of IPF infection secondary to influenza virus infection and further explore its etiologic mechanism and high-risk factors.

**Methods:**

All adult patients with confirmed influenza A (H1N1) virus infection admitted to the intensive care units (ICUs) of Nanjing Drum Hospital from November 2017 to March 2018 were retrospectively selected. The differences in baseline factors, risk factors, immune function and outcome parameters were studied between patients with and without IPF.

**Results:**

Of the 19 critically ill patients with H1N1 infection, 11 (57.9%) developed IPF infection after 7 days of ICU admission. Two patients had proven and nine probable IPF infection. A difference in human leukocyte antigen–DR isotype (△HLA-DR; day 7–day 1) was found between the two groups. △HLA-DR (day 7–day 1) was higher in patients with no IPF infection than in those with IPF infection [(14.52 ± 14.21)% vs ( − 11.74 ± 20.22)%, *P* = 0.019]. The decline in HLA-DR indicated impaired immune function secondary to fungal infection in patients with H1N1 infection.

**Conclusions:**

IPF infection was diagnosed in 57.9% of critically ill patients with H1N1 virus infection after a median of 7 days following ICU admission. A continuous decline in immune function could lead to the development of IPF infections. Dynamic monitoring of immune function may help in the early detection of IPF infection.

## Introduction

Since the first case of influenza A (H1N1) virus infection found in Mexico in 2009, the virus has spread worldwide and resulted in pandemics [1]. Nearly 60.8 million were infected with H1N1from April 2009 to April 2010; of these, 274,304 were hospitalized and 12,469 died in the USA [2, 3]. The H1N1 infection was usually complicated by several diseases including acute respiratory distress syndrome, cardiac disorders, bacterial pneumonia and others. Pneumonia was one of the most common complications. Influenza and pneumonia mortality accounted for 2.2% and 2.0%, becoming the eighth and ninth causes of death in 2009 and 2010 in the USA, respectively [4, 5]. Recent studies also revealed that 18–61% of hospitalized patients with H1N1 infection had pneumonia characteristics, demanding more attention to influenza-induced pneumonia [6–8].

Previous studies have shown that severe pneumonia caused by influenza virus infection can be secondary to invasive pulmonary fungal (IPF) infection. It is probably because these people have predisposing factors, such as lung disease, malignancy and chronic obstructive pulmonary disease, affecting the function of T lymphocytes, macrophages and neutrophils, or influenza increases susceptibility to bacterial super-infection by altering defensive mechanisms [9, 10]. Once it occurs, treatment decisions change and the prognosis is greatly different [11]. However, due to the limited case reports in recent years, the robust data on the prevalence, pathogenesis, risk factors and final prognosis of the aforementioned infection are sparse [12–14]. In a retrospective study published in 2012, the authors recruited 40 critically ill patients with H1N1 infection and nine of them developed invasive pulmonary aspergillosis, accounting for 23% [15].

**Fig. 1:**
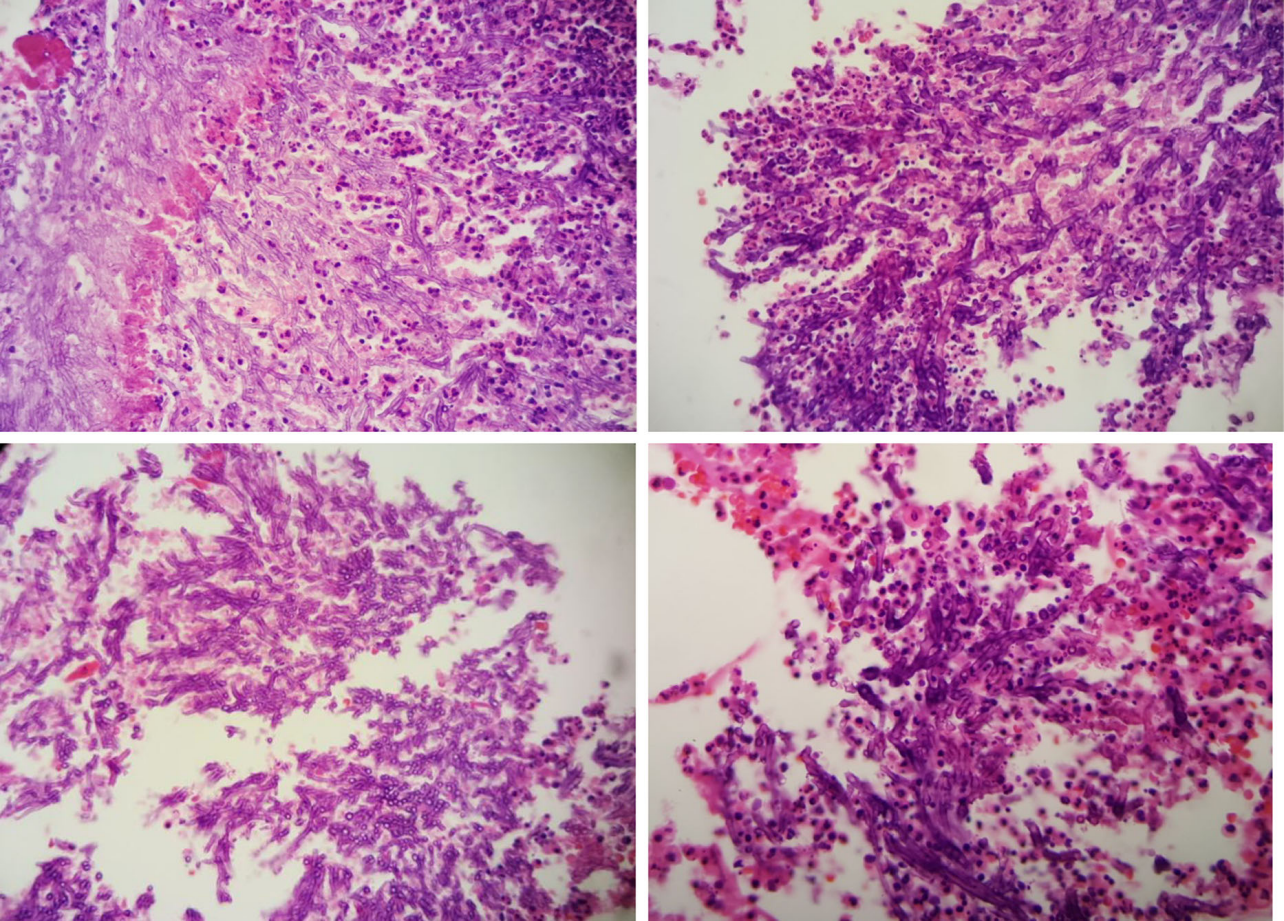
Pathological examination of the airway mass from patients 6 and 7 revealed fibrous necrotic tissue and fungal filaments, which confirmed a fungal infection (× 100)

This study included 19 cases of severe pneumonia caused by influenza virus needing treatment and monitoring at the intensive care unit (ICU) in the last 4 months to provide more data for the H1N1-complicated IPF infection. In this retrospective study, the incidence of invasive fungal infection secondary to influenza virus was summarized and its etiology mechanism and high-risk factors were further explored (Fig. 1).

## Materials and Methods

### Study Population

This study was performed at the adult ICUs of Nanjing Drum Hospital. The medical records of all the patients with H1N1 virus infection during or 1 week prior to the ICU stay from November 2017 to March 2018 were retrospectively reviewed. The exclusion criteria were as follows: (a) age < 18 or ≥ 80 years; (b) had primary immunodeficiency disease, autoimmune disease or malignancy; (c) received hormones or immunosuppressive agents for nearly 3 months or surgical intervention; and (d) died within 14 days of hospitalization.

The diagnosis of H1N1 virus infection was based on a positive result in a probe-based reverse transcription polymerase chain reaction test for H1N1 from a nasopharyngeal swab or bronchoalveolar lavage (BAL) fluid. This observational retrospective study without any specific intervention was reviewed and approved by the hospital’s institutional review boards. All data were processed anonymously.

## Definitions

IPF infection was confirmed following the revised European Organization for Research and Treatment of Cancer/Invasive Fungal Infections Cooperative Group and the National Institute of Allergy and Infectious Diseases Mycoses Study Group (EORTC/MSG) guidelines.

**Fig. 2:**
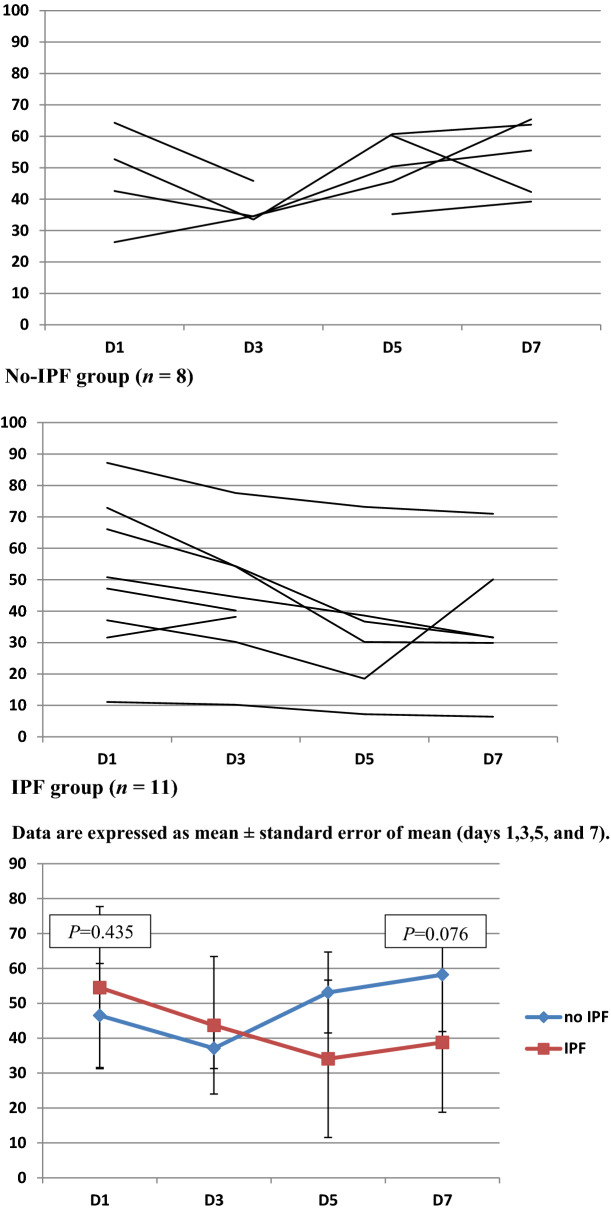
Time course of HLA-DR expression (%) in the IPF and no-IPF groups after admission

The patients had proven IPF infection if the microscopic evidence of dichotomous branching hyphae with a positive culture for fungi from endobronchial biopsy (not BAL) was obtained, irrespective of host factors or clinical features (Fig. 2).

Probable IPF infection required a host factor, clinical features and mycological evidence of fungi. The mycological evidence was based on microscopy or culture of fungi from a BAL specimen or a galactomannan (GM) optical index (0.5 from a BAL or serum sample). The sandwich enzyme-linked immunosorbent assay for GM detection was performed according to the manufacturer’s protocol. Lower tract respiratory samples were inoculated onto a Sabouraud agar (2 days at 37 °C and another 19 days at 30 °C) for fungal isolation. Fungal species were identified by their culture characteristics and morphologies.

The severity of illness was assessed using the acute physiology and chronic evaluation (APACHE) II score and the sequential organ failure assessment (SOFA) score after 24 h of admission.

The doses and frequency of all drugs administered in the month before and during admission to the ICU were updated in the medical records.

Biochemical data, including CD4 + /CD8 + ratio, percentage of natural killer (NK) and B cells and human leukocyte antigen–DR isotype (HLA-DR) in peripheral blood mononuclear cells, were recorded. Peripheral venous blood samples (2 mL) were obtained from patients on days 1, 3, 5 and 7 after admission using a strict aseptic technique. Biochemical data were determined using the BD Multitest Identification Mark Kit (BD Corporation, USA, state code 340503). The other reagents and equipment used were as follows: fluorescein isothiocyanate antihuman CD14 (eBioscience Corporation, USA, state code IM1638U), phycoerythrin (PE) antihuman HLA-DR (eBioscience Corporation), PE mouse immunoglobulin G (IgG)2b isotype control (eBioscience Corporation, state code IM0464U), FACSort flow cytometer (BD Corporation) and CellQuest software.

### Statistical Analysis

The SPSS22.0 software was used for data analysis. The quantitative data with normal distribution and homogeneity of variance were expressed as mean ± standard deviation (SD) and compared using the independent-sample *t* test. The groups were compared using the nonparametric Wilcoxon test. The qualitative data were expressed as a percentage. The groups were compared using the chi-square test. The test level was 0.05.

## Results

### Patient Characteristics

The baseline factors are listed in Tables 1 , 2, 3. A total of 19 adult critically ill patients with confirmed H1N1 infection were included in the study between November 2017 and March 2018. Table 1 shows that the patients had a mean age of 49.18 ± 14.38 years, and 13 patients (68.4%) were male. They were divided into two groups based on the occurrence of secondary fungal infections in the course of the disease: IPF and no IPF. The initial symptoms, body temperature, leukocyte count and neutrophil count were not different between the two groups (Table 2). Also, no difference was observed in the onset to visiting time, aggravation time or ICU time (Table 3). Four patients (23.5%) received corticosteroids before admission to hospital as therapy for “severe acute respiratory distress syndrome.” All patients received antiviral therapies such as oseltamivir and peramivir. Although the initial antiviral time seemed longer in the IPF group (10.10 ± 2.29) than in the no-IPF group (6.86 ± 2.12), the difference was not significant (*P* = 0.272). On admission, the mean APACHE II score was 17.06 ± 6.81 and the median SOFA score was 5.35 ± 3.08, reflecting a high severity of illness and a high incidence of multiple organ dysfunction syndrome. During hospitalization, the worst oxygenation index of patients was 93.34 ± 30.57 mm Hg. The respiratory support methods included extracorporeal membrane oxygenation (ECMO), tracheal intubation, noninvasive ventilation, high-flow nasal cannulae and oxygen therapy.

**Table 1:** Demographic and clinical characteristics of the patients included in the study

	Total (*n* = 19)	No IPF (*n* = 8)	IPF (*n* = 11)	*P*
Age (year)	49.2 ± 14.4	44.4 ± 12.2	52.5 ± 15.4	0.268
Sex (male), n (%)	13/19 (68.4%)	4/8 (50%)	9/11 (81.8%)	
*Contact history*				
None	17/19	7/8	10/11	
Poultry	2/19	1/8	1/11	
*Occupation*				
Farmer	6/19	2/8	4/11	
Unemployed	7/19	4/8	3/11	
Other	6/19	2/8	4/11	
*Comorbid illness*				
Healthy	10/19	4/8	6/11	
Hypertension	5/19	1/8	4/11	
Liver disease	4/19	4/8	0/11	
COPD (not taken systemic corticosteroids)	1/19	1/8	0/11	
Diabetes mellitus	2/19	0/8	2/11	
Coronary heart disease	4/19	1/8	3/11	
Atrial fibrillation	1/19	0/8	1/11	
Cerebellar hypoplasia	1/19	1/8	0/11	
Cerebral infarction	2/19	1/8	1/11	
Parkinson’s disease	1/19	1/8	0/11	
Pregnancy, *n* (%)	3/19 (15.8%)	2/8 (25%)	1/11 (9.1%)	
Obesity (BMI ≥ 30 kg/m^2^), *n* (%)	1/19 (5.3%)	1/8 (12.5%)	0/11 (0%)	
Worst oxygenation index (mm Hg)	93.34 ± 30.57	103.29 ± 24.03	85.61 ± 34.14	0.265
*Respiratory support*				
None, *n* (%)	3/19 (15.8%)	1/8 (12.5%)	2/11 (18.2%)	
ECMO, *n* (%)	5/19 (26.3%)	3/8 (37.5%)	2/11 (18.2%)	
ECMO, days	9 (1–33)	9 (8–10)	17 (1–33)	
MV, *n* (%)	5/19 (26.3%)	2/8 (25%)	3/11 (27.3%)	
NIV, *n* (%)	5/19 (26.3%)	2/8 (25%)	3/11 (27.3%)	
Mechanical ventilation, days	9 (0–45)	10 (0–17)	8 (0–45)	
HFNC, *n* (%)	1/19 (5.3%)	0/8 (0%)	1/11 (9.1%)	
Vasopressors, *n* (%)	8/19 (42.1%)	3/8 (37.5%)	5/11 (45.5%)	
Vasopressor (day)	0.2 (0–40)	0 (0–6)	0.5 (0–40)	
Renal replacement therapy, *n* (%)	2/19 (10.5%)	0/8 (0%)	2/11 (18.2%)	
Renal replacement therapy (day)	0 (0–60)	0(0)	0 (0–60)	
APACHE II	17.1 ± 6.8	19.0 ± 9.0	15.7 ± 4.89	0.341
SOFA	5.4 ± 3.1	4.4 ± 2.4	6.0 ± 3.2	0.316

*APACHE* acute physiology and chronic evaluation score, *BMI* body mass index, *COPD* chronic obstructive pulmonary disease, *ECMO* extracorporeal membrane oxygenation, *HFNC* high-flow nasal cannula, *IPF* invasive pulmonary fungus, *MV* mechanical ventilation days, *NIV* noninvasive mechanical ventilation, *SOFA* sequential organ failure assessment

**Table 2:** Initial clinical symptoms, previous treatment and prognosis of the patients

	Total (*n* = 19)	No IFI (*n* = 8)	IFI (*n* = 11)	*P*
*First symptoms*				
Cough	19/19	8/8	11/11	
Expectoration	12/19	5/8	7/11	
Sore throat	2/19	2/8	0/11	
Wheezing or dyspnea	2/19	1/8	1/11	
Headache	1/19	0/8	1/11	
Initial body temperature (°C)	39.1 ± 0.7	39.0 ± 0.8	39.2 ± 0.6	*0.58*
Initial leukocyte (× 10^9^/L)	6.9 ± 3.2	7.5 ± 3.9	6.3 ± 2.4	*0.466*

**Table 3:** Previous treatment of the patients

	Total (*n *= 19)	No IFI (*n *= 8)	IFI (*n *= 11)	*P*
Onset to visiting time	3.7 ± 5.9	3.1 ± 2.3	4.0 ± 7.5	*0.777*
Onset to aggravation time	6.6 ± 5.2	5.0 ± 2.3	7.7 ± 6.4	*0.306*
Onset to ICU time	9.1 ± 6.2	6.3 ± 2.9	11.0 ± 7.3	*0.128*
Use of glucocorticoids in the course of disease	4/19	2/8	2/11	
Initial antiviral therapy				
Oseltamivir	15/19	5/8	10/11	
Peramivir	4/19	3/8	1/11	
Time of initial antiviral	8.8 ± 5.8	6.9 ± 2.1	10.1 ± 2.3	*0.272*
Time of virus clearance	10.9 ± 7.8	12.3 ± 9.0	9.7 ± 6.9	*0.514*

Onset to visiting time: Onset from first influenza symptoms to outpatient visit.

Onset to aggravation time: Patient's symptoms cannot be tolerated.

Time of initial antiviral treatment: Onset of symptoms to start antiviral treatment.

Time of virus clearance: Time for virus to turn negative [The diagnosis of H1N1 virus infection was based on a positive result in a probe-based reverse transcription polymerase chain reaction test for H1N1 from a nasopharyngeal swab or bronchoalveolar lavage (BAL) fluid.]

## Patient Prognosis

The results are shown in Table 4. In the present study, 15/19 (78.9%) of patients, 7 in the IPF group and 8 people in the no-IPF group, were still alive at ICU discharge and hospital discharge. The length of ICU stay and hospital stay was 25.29 ± 18.04 days and 25.94 ± 17.70 days, respectively. The two groups had no significant difference in the length of ICU stay or hospital stay (*P* = 0.293 or 0.200).

**Table 4:** Prognosis of the patients

	Total (*n *= 19)	no IFI (*n *= 8)	IFI (*n *= 11)	*P*
Alive at ICU discharge, n (%)	15/19 (78.9%)	7/8 (87.5%)	8/11 (72.7%)	*0.334*
Alive at hospital discharge, n (%)	15/19 (78.9%)	7/8 (87.5%)	8/11 (72.7%)	*0.521*
Length of ICU stay	25.3 ± 18.0	24.6 ± 11.9	25.8 ± 22.0	*0.293*
Length of hospital stay	26.0 ± 18.0	26.1 ± 10.5	25.8 ± 22.0	*0.2*

Onset to visiting time: Onset from first influenza symptoms to outpatient visit.

Onset to aggravation time: Patient’s symptoms cannot be tolerated.

Time of initial antiviral treatment: Onset of symptoms to start antiviral treatment.

Time of virus clearance: Time for virus to turn negative [The diagnosis of H1N1 virus infection was based on a positive result in a probe-based reverse transcription polymerase chain reaction test for H1N1 from a nasopharyngeal swab or bronchoalveolar lavage (BAL) fluid.]

## IPF Infection

The study found that 57.9% (11/19) of the critically ill patients with H1N1 infection developed IPF infection during ICU stay (Table 5). The time between diagnosis of influenza and IPF infection ranged from 4 to 12 days, with a mean of 7.3 days and a median of 7 days. On the basis of the modified EORTC/MSG criteria, IPF infection probably occurred in nine patients and was confirmed in two patients. Nine patients were treated with antifungal therapy. All of them were treated with voriconazole.

**Table 5:** Overview of all individual cases of IPF infection

Patient	Fungus	GM blood (pg/mL)	GM blood	GM BAL	Day of first indication of IPF infection after ICU admission (days)	Diagnosis of IPF infection	Drug
1	*Aspergillus niger*, aflatoxin	93.1	0.14		7	Probable	Voriconazole
2	*Candida* *Albicans*	104.8	0.32		10	Probable	Voriconazole
3	*Aspergillus*	24	0.98		4	Probable	Voriconazole
4	*Aspergillus*, *Candida albicans*	111.3	0.51	3.9	12	Probable	Voriconazole
5	*Aspergillus*	97.7	0.17		6	Probable	Voriconazole
6	*Aspergillus* *Fumigatus*	188	4.74	4.89	7	Proven	Voriconazole
7	*Candida* *Albicans*	301.2	2.31		5	Proven	Voriconazole
8	*Candida kurou*	17	0.06		6	Probable	None
9	*Candida* *Albicans*	31.2	0.09		4	Probable	None
10	*Candida* *Albicans*	312.6	0.31	0.98	10	Probable	Voriconazole
11	*Aspergillus*	9.8	0.46	0.51	10	Probable	Voriconazole

## Immune Function Monitoring

Table 6 shows that the HLA-DR percentage on days 1 and 7 did not differ significantly between the groups. However, a difference in △HLA-DR (day 7–day 1) was found between the two groups (*P* = 0.019). Also, differences in the NK cell count were observed on day 1 between the two groups. The CD4 + /CD8 + ratio, percentage of B cells, percentage of neutrophils and percentage of lymphocytes did not differ significantly between the groups at any of the time points.

**Table 6:** Immune function monitoring

	Total (*n* = 19)	No IPF (*n* = 8)	IPF (*n* = 11)	*P*
HLA-DR day 1 (%)	51.2 ± 20.1	46.5 ± 14.9	54.5 ± 23.2	0.435
HLA-DR day7 (%)	47.1 ± 20.5	58.2 ± 16.3	38.76 ± 20.0	0.076
△HLA-DR (day 7–day 1) (%)	–0.50 ± 21.9	14.5 ± 14.2	–11.7 ± 20.2	0.019
CD4 + /CD8 + day 1	1.5 ± 0.6	1.3 ± 0.6	1.7 ± 0.5	0.293
CD4 + /CD8 + day 7	1.7 ± 1.0	1.3 ± 1.0	1.9 ± 1.0	0.757
△CD4 + /CD8 + (day 7 – day 1)	0.1 ± 0.8	0.0 ± 0.6	0.2 ± 1.0	0.711
NK cell day 1 (× 10^9^/L)	0.1 ± 0.1	0.1 ± 0.1	0.1 ± 0.1	0.950
NK cell day 7 (× 10^9^/L)	0.1 ± 0.1	0.2 ± 0.1	0.1 ± 0.1	0.372
△NK cell(day 7 – day 1) (× 10^9^/L)	0.0 ± 0.1	0.1 ± 0.1	–0.0 ± 0.1	0.066
B cell day 1(× 10^9^/L)	0.2 ± 0.1	0.2 ± 0.1	0.2 ± 0.1	0.997
B cell day 7(× 10^9^/L)	0.2 ± 0.1	0.2 ± 0.1	0.2 ± 0.1	0.776
△B cell(day 7 – day 1) (× 10^9^/L)	0.0 ± 0.1	0.0 ± 0.2	0.0 ± 0.1	0.260
Neutrophils day 1(× 10^9^/L)	5.5 ± 3.4	5.4 ± 4.1	5.6 ± 3.0	0.414
Neutrophils day 7(× 10^9^/L)	8.0 ± 4.1	9.3 ± 4.4	7.1 ± 3.9	0.824
△Neutrophils(day 7 – day 1) (× 10^9^/L)	2.6 ± 3.2	3.8 ± 4.0	1.5 ± 2.1	0.135
Lymphocyte day 1(× 10^9^/L)	0.6 ± 0.3	0.5 ± 0.2	0.7 ± 0.4	0.122
Lymphocyte day 7(× 10^9^/L)	1.1 ± 0.5	1.0 ± 0.5	1.2 ± 0.5	0.435
△Lymphocyte (day 7–day 1) (× 10^9^/L)	0.5 ± 0.5	0.5 ± 0.6	0.5 ± 0.4	0.168

## Discussion

The intensive understanding of H1N1 virus, application of vaccines and improvements in treatment options have facilitated the entry into the post-pandemic period [16]. Although patients with H1N1 infection in post-pandemic years were less serious than those in the pandemic year, some complications are still common, severe and fatal. A potential complication of influenza is super-infection with fungal species. An increasing number of cases have been reported over time [17–19].

Wauters et al. [15] retrospectively selected all adult patients with confirmed H1N1 virus infection admitted to the ICU of the two tertiary care hospitals from September 2009 to March 2011. These 40 patients had an average APACHE II score of 23 ± 8, an average SOFA score of 11 and a high degree of disease risk. Of 40 critically ill patients with confirmed H1N1, 9 (23%) developed IPA after 3 days of ICU admission. In contrast, the present study focused on recruiting patients from the department within 4 months, indicating that the virus was more homologous and the differences between individuals diagnosed and treated were small. The average APACHE II score was 17.06 ± 6.81, and the SOFA score was 5.35 ± 3.08. The severity of the disease and organ failure appeared to be less than that reported by Wauter. However, further observations revealed that the patients in the present study had a relatively short period of time from onset to ICU admission, and the statistics included APACHE II scores and SOFA scores within 24 h of ICU admission. After ICU admission, the disease was still in a developing stage and did not reach the critical peak in 24 h. The lowest oxygenation index was 93.34 ± 30.57 mm Hg in the course of the disease. Also, 88% of patients opted for mechanical ventilation and 28% for ECMO. In the study by Wauter, 23.5% of patients opted for ECMO and 88.2% for mechanical ventilation. Hence, the patients in the two studies were quite similar in terms of criticality and disease characteristics. In the aforementioned study, the incidence of invasive fungal infection in the lungs was 9/40 (23%). In the present study, the incidence was 11/19 (58.9%). One possible hypothesis was the evolution of more virulent influenza strains, as exemplified by the pandemic H1N1 strain, causing more severe pneumonia. Compared with previous findings [17], the average viral transposition time of the patients was longer [(10.88 ± 7.81) days] and the duration of the disease increased in the present study. This result also proved the previous hypothesis. A retrospective study found that the risk of IPF infection was higher when respiratory viruses were in circulation, in particular, influenza A [20]. IPF infections were prone to occur during viral infection and replication process. The time between diagnosis of influenza and IPF infection in 11 patients was a mean of 7.3 days (SD ± 0.7). This result was almost consistent with previous findings [21].

The data in the present study suggested that the use of corticosteroids 7 days before ICU admission was an independent risk factor for fungal super-infection. In this study, no-IPF group had prehospitalization rates of 28.6% and 20%. No significant difference was observed between the two groups, demonstrating no significant relationship between glucocorticoid use and secondary fungal infection before hospital admission. This result was not consistent with previous observations.

Therefore, other mechanisms leading to decreased immune function might exist. Influenza virus affects the production of cytokines, in turn impacting the immune system. H1N1 virus leads to an increased and prolonged production of interleukin-10 (IL-10) in plasma. This, in turn, affects Th2 response and adaptive immunity mediated by Th2, which is associated with an increased risk of IPF infection [22]. The influenza virus has been reported to impair phagocytosis and alveolar macrophage function, increasing the risk of IPF infection [9]. The severe systemic inflammatory syndrome, which is called compensatory anti-inflammatory response syndrome or immunoparalysis, may occur in patients with severe influenza infection, making them vulnerable to severe super-infections by bacteria and fungi [10].

The immune function indicators of patients were evaluated within 7 days after admission, and the dynamic changes in immune function were observed. In previous studies, a decrease in the peripheral blood lymphocyte count was one of the risk factors for secondary fungal infections [23]. However, this phenomenon was not observed in the present study. Interestingly, the HLA-DR percentage on days 1 and 7 did not differ significantly between the groups. Therefore, the difference in HLA-DR percentage on days 1 and 7 was compared between the two groups in the present study. △HLA-DR (day 7–day 1) was higher in patients with no IPF infection than in those with IPF infection. The decline in HLA-DR indicated impaired immune function secondary to fungal infection in patients with influenza A/H1N1 infection. With constant exposure to fungi, the normal human immune system developed defenses for fungal clearance via alveolar macrophages, neutrophils, monocytes and NK cells [24]. This study showed that a continuous decline in immune function could lead to the development of IPF infections. In previous studies, most patients with aspergillosis associated with influenza did not have classic risk factors or radiographic findings [25]. Therefore, dynamic monitoring of immune function may help in the early detection of IPF infection.

## Conclusions

In summary, IPF infection is a potential complication of severe influenza infection. Progressive pulmonary infection after the initial diagnosis of influenza should raise suspicion of super-infection, including with fungi.

This retrospective study had three limitations. First, the lower percentage of histological examinations under bronchoscopic biopsy might have resulted in missed diagnosis in some patients. Second, only 19 patients were identified and the sample size was insufficient to investigate all the features. Finally, how to enhance immune function and its impact on fungal infections need further investigation.

## Availability of Data and Materials

The dataset used and analyzed in the present study is available from the corresponding author on reasonable request.

## Abbreviations

APACHE II score: Acute physiology and chronic evaluation II score
ARDS: Acute respiratory distress syndrome
BAL: Bronchoalveolar lavage fluid
CRRT: Continuous renal replacement therapy
ECMO: Extracorporeal membrane oxygenation
GM: Galactomannan
HFNC: High-flow nasal cannulae
HLA-DR: Human leukocyte antigen–DR isotype
ICU: Intensive care unit
IPF: Invasive pulmonary fungal
MODS: Multiple organ dysfunction syndrome
SOFA: Sequential organ failure assessment
